# The soluble biomarker calprotectin (a S100 protein) is associated to ultrasonographic synovitis scores and is sensitive to change in patients with rheumatoid arthritis treated with adalimumab

**DOI:** 10.1186/ar3503

**Published:** 2011-10-26

**Authors:** Hilde Berner Hammer, Magne K Fagerhol, Tale Norbye Wien, Tore K Kvien

**Affiliations:** 1 Dept of Rheumatology, Diakonhjemmet Hospital, Box 23, 0319 Oslo, Norway; 2 Blood Bank and Dept of Immunology, Oslo University Hospital, Ullevål Hospital, Kirkeveien 166, 0450 Oslo, Norway; 3 Dept of Internal Medicine, Oslo University Hospital, Rikshospitalet, Sognsvannsveien 20, 0372 Oslo, Norway

**Keywords:** Calprotectin, acute phase proteins, rheumatoid arthritis, ultrasonography, anti-TNF treatment

## Abstract

**Introduction:**

Calprotectin (MRP8/MRP14, S100A8/A9) is associated with disease activity in patients with rheumatoid arthritis (RA). Ultrasonography (US) is a reliable method for evaluation of synovitis (B-mode (BM) and power Doppler (PD)). The present objectives were to explore in RA patients the associations between calprotectin and a comprehensive US examination, as well as the responsiveness of calprotectin compared to other inflammatory markers during anti-TNF treatment.

**Methods:**

A total of 20 RA patients starting treatment with adalimumab were examined longitudinally by US (BM and PD (semi-quantitative scores 0 to 3) of 78 joints, 36 tendons/tendon groups and 2 bursae) and clinically at baseline and after 1, 3, 6 and 12 months. Associations between the US sum scores and the inflammatory markers calprotectin, serum amyloid A (SAA), CRP and ESR were explored by correlation and linear regression analyses, and the response to treatment was assessed by Standardized Response Mean (SRM).

**Results:**

The inflammatory markers, clinical examinations and US sum scores improved during treatment (*P *< 0.001). Of the inflammatory markers, calprotectin had the highest correlation coefficients with the total BM and PD sum scores (median (range) 0.59 (0.37 to 0.76) for BM and 0.56 (0.38 to 0.72) for PD). Even higher correlations were found between calprotectin and sum US scores of reduced number of joint counts. Calprotectin made a considerable contribution to total US sum scores in the linear regression analyses (*P *= 0.001 to 0.031) and among the inflammatory markers, calprotectin had the highest SRM (0.84 at one month).

**Conclusions:**

Calprotectin was associated with the sum scores from a comprehensive US assessment and was responsive to change during anti-TNF treatment. Thus, examination of this leukocyte protein could be of additional value in the assessment of RA patients on biologic treatment.

## Introduction

Assessment of inflammatory activity is of pivotal importance for the optimal treatment of patients with rheumatoid arthritis (RA). Up till now the erythrocyte sedimentation rate (ESR) and the acute phase protein C-reactive protein (CRP) are the most widely used laboratory markers for evaluation of inflammatory activity in patients with RA. Serum amyloid A (SAA) (an apo-lipoprotein associated with high-density lipoprotein in plasma) is produced in the liver after stimulation by the pro-inflammatory cytokines IL-1, IL-6 and TNF-alpha, and 1, 000-fold increases may be found during severe inflammation [1]. Thus, it acts as an acute phase protein like CRP, but may be more sensitive to inflammation [2].

Calprotectin is a major leukocyte protein, constituting 40 to 60% of the cytosolic protein in neutrophile granulocytes as well as being a major monocyte/macrophage protein [3-5]. Calprotectin (also named MRP-8/MRP-14 [6] and S100A8/A9 [7]) is a calcium-binding S100 protein [8] and it is one of the damage-associated molecular pattern molecules (DAMPs) highly up-regulated in various autoimmune disorders [9]. The protein is released during activation and turnover of leukocytes [10] and calprotectin levels are strongly associated with disease activity in RA patients [11,12]. Concentrations of calprotectin are high in synovial fluid in RA patients in contrast to low levels in osteoarthritis patients [13] and calprotectin is associated to radiographic damage and is a predictor of radiographic progression in RA patients [12,14].

Ultrasonography (US) is a valid and reliable tool for detecting synovitis in patients with RA [15-17], and joints, tendons and bursae may be examined. The extent of synovitis is usually scored semi-quantitatively (scale 0 to 3) both for grey scale (or B-mode (BM)) synovitis (including combined evaluation of synovial hypertrophy and effusion) and power Doppler (PD) vascularization, and the US scores have been shown to be sensitive for improvement during biological treatment in RA patients [17-20]. A recently published study from our group showed a high degree of reliability when we used a similar scoring system as presently applied [21]. There is no consensus on the optimal number of joints and tendons to be assessed for US evaluation of inflammatory activity in RA patients, but similar sensitivity to change has been found for US examinations of different combinations of joints and tendons during biological treatment [22].

The objectives of the present study were to examine the associations between the levels of calprotectin and a comprehensive as well as reduced US joint scores and to explore the responsiveness of calprotectin compared to other inflammatory markers during biologic treatment in patients with RA.

## Materials and methods

### Patients

The present study is part of a work described in detail previously [23]. In short, a comprehensive US assessment was performed in 20 patients with RA [24] (median (range) age 53 (21 to 78) years, disease duration 7.5 (1 to 26) years, 15 women, 70% IgM rheumatoid factor positive) the same day as they started treatment with adalimumab and after 1, 3, 6 and 12 months. Adalimumab (as their first biological medication) was dosed 40 mg every other week, all patients were on methotrexate as co-medication, 14 patients used additional prednisolone (median (range) dose 7.5 (3.75 to 15) mg) and two patients were on daily non-steroidal anti-inflammatory drug.

The patients gave written consent according to the Declaration of Helsinki, and the study was approved by the local ethics committee (the regional committee for medical and health research ethics (REK), South-East).

### Laboratory examinations

The traditional inflammatory markers included ESR and CRP (both analyzed by use of in-house standard methodology, with upper normal levels of 20 mm/h for ESR and 4 mg/l for CRP). Serum and EDTA plasma from all the five examinations were frozen at ÷70°C for assessment of SAA and calprotectin after completion of the clinical study. SAA was measured in serum by immunonephelometry using an N-Latex SAA reagent (Dade Behring Inc., Marburg, Germany) with normal levels ≤ 6.4 mg/l [25]. All plasma samples were analyzed for calprotectin at the same time by use of ELISA as described previously (using kits from Calpro AS, Norway) [13,26], with normal levels ≤ 0.91 mg/l. Assessments of calprotectin in plasma have shown coefficients of variation of 5% within and 13% between assays [27].

### Clinical evaluations

One of two study nurses, with more than five years experience with joint counts in clinical studies, assessed 40 joints for tenderness and swelling (proximal interphalangeal 1 to 5, metacarpophalangeal 1 to 5, wrist, elbow, shoulder, knee, ankle and metatarsophalangeal 1 to 5) and they scored the global disease activity caused by RA on a VAS scale (assessor's global VAS). They were blinded for the results of the US examinations. Patient's evaluation of disease activity was assessed (VAS), and DAS28 [28] was calculated.

### Ultrasonography

All patients were assessed by extensive US examinations (performed by one experienced sonographer, HBH) with use of a 5 to 13 MHz probe (Siemens Antares, Sonoline, Siemens Medical Solutions, 1230 Shorebird Way Mountain, CA, USA) with fixed settings optimal for power Doppler signals in more superficial joints. The joints assessed bilaterally by use of standard projections [29] included proximal interphalangeal 1 to 5, metacarpophalangeal 1 to 5, carpometacarpal 1 to 5, wrist (radiocarpal, intercarpal and radioulnar joints), elbow, shoulder (glenohumeral and acromioclavicular joints) hip, knee, ankle (talocrural joint), 4 major foot joints (talonavicular, subtalar, calcaneocuboidal and cuneonavicular joints), tarsometatarsal 1 to 5, metatarsophalangeal 1 to 5 and the interphalangeal joint of the first toe (a total of 78 joints), in addition to tendons bilaterally in the wrists (at the level of the radiocarpal joint: all the 6 compartments dorsally and the flexor digitorum superficialis and profundus, flexor pollicis longus and flexor carpi radialis tendons at the palmar side), in the ankles (at the level of the malleol and distal tibia; medial, anterior and lateral tendon groups) as well as the long biceps tendon (a total of 36 tendons or tendon groups) and subdeltoid bursa bilaterally. The arthritis, tenosynovitis and bursitis were all scored for BM presence of synovial hypertrophy and fluid (combined) and presence of vascularization (PD) on a 4-point scale: 0 = none, 1 = minor, 2 = moderate or 3 = major presence. The US examiner was blinded for US results from the previous examinations as well as for the results from the clinical joint assessments and laboratory tests performed the same day.

### Statistics

Wilcoxon signed rank test was used to examine changes from baseline of the US, clinical and laboratory results during follow-up. Correlations were analyzed by use of Spearman's rank correlations. Linear regression analysis was performed for all examinations with total sum score BM or sum score PD as the dependent variable with age, sex, disease duration and DAS28 as well as each of the biomarkers (one at a time) as the independent variables. A *P*-value < 0.05 was considered statistically significant. The responsiveness to change was explored by calculation of the Standardized Response Mean (SRM) as the mean change divided by the SD of the change (definitions of SRM results: < 0.50 = small, 0.50 to 0.80 = moderate and > 0.80 = large responses).

## Results

**Figure 1:**
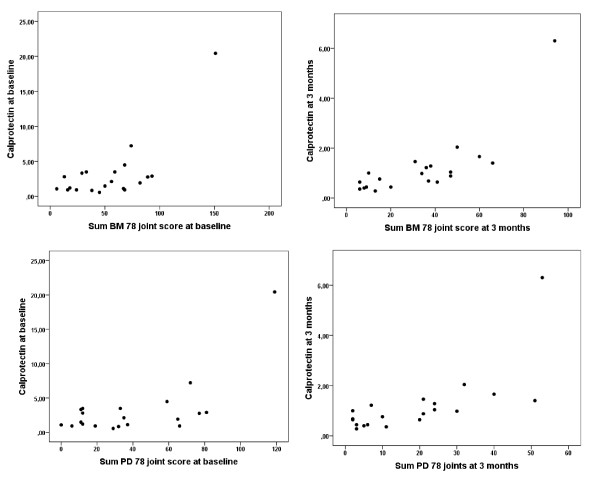
**Scatterplots of calprotectin levels with corresponding sum B-mode (BM) or power Doppler (PD) scores**. The scatterplots illustrate the distribution of the calprotectin levels with the corresponding total sum B-mode (BM) or power Doppler (PD) scores at baseline and at three months examination.

The laboratory, clinical and US results improved significantly during the study (Table 1). Significant cross-sectional correlations were found between the inflammatory biomarkers and both BM and PD total sum scores at most of the examinations, with calprotectin having the highest correlation coefficients (Table 2). Figure 1 illustrates the associations between calprotectin and total sum US results at baseline and after three months. Of the correlation analyses between calprotectin levels and US sum scores of different reduced numbers of joints and tendons (the 7-, 12-, 28- and 44-joint scores [22]), the 12-joint score was found to have the highest correlation coefficients at different examinations (median (range) 0.59 (0.53 to 0.83) (*P *= 0.02 to < 0.001) for BM and 0.58 (0.42 to 0.72) (*P *= 0.06 to < 0.001) for PD).

**Table 1:** Median (range) of the laboratory, clinical and US scores

	Baseline	1 month	3 months	6 months	12 months
Calprotectin, mg/l (normal ≤ 0.9l)	2.02 (0.56 to 20.44)	0.92 (0.32 to 18.86)**	0.93 (0.28 to 6.30)**	0.94 (0.24 to 22.02)**	1.08 (0.36 to 17.60)
SAA mg/l (normal ≤ 6.4)	10.60 (2.17 to 300)	6.94 (1.37 to 270)*	4.19 (0.70 to 38.5)**	4.39 (1.61 to 176)*	8.37 (1.12 to 136)
CRP, mg/l (normal ≤ 4)	4.5 (1 to 136)	2.0 (1 to 163)	2.5 (1 to 26)**	2.5 (1 to 78)	4.5 (1 to 86)
ESR, mm/h (normal ≤ 20)	20 (3 to 67)	13 (3 to 108)**	14.5 (2 to 65)**	14.5 (5 to 99)	17.5 (3 to 78)
DAS28	5.32 (3.40 to 7.70)	4.20 (1.56 to 8.90)**	4.26 (0.83 to 7.02) **	4.36 (1.59 to 7.58)**	4.33 (1.40 to 6.60)**
Assessors VAS	26 (6 to 55)	15 (4 to 52)*	14 (3 to 40)**	11 (2 to 57)	10 (3 to 44)**
No of swollen joints (of 40)	8 (1 to 19)	5 (0 to 22)	6 (0 to 16)**	4 (0 to 16)*	4 (0 to 14)**
No of tender joints (of 40)	12 (0 to 23)	9 (0 to 35)	7 (0 to 16)**	6 (0 to 16)**	7 (0 to 21)**
BM score	53 (6 to 151)	39 (3 to 121)**	35 (6 to 94)**	24 (0 to 86)**	27 (1 to 96)**
PD score	33 (0 to 119)	21 (0 to 83)**	16 (2 to 53)**	14 (0 to 76)**	13 (0 to 72)**

*P < 0.05, **P < 0.01 (Wilcoxon signed rank test for changes from baseline to the different follow-up examinations).

BM, B-mode; CRP, C-reactive protein; DAS28, Disease Activity Score 28 joints; ESR, erythrocyte sedimentation rate; PD, power Doppler; SAA, serum amyloid A; Assessors VAS, assessors global disease activity score on a visual analogue scale (0 to 100).

**Table 2:** Spearman's rank correlations between the laboratory variables and sum B-mode/power Doppler scores

	Baseline	1 month	3 months	6 months	12 months
**Calprotectin**	0.45*/0.38	0.37/0.53*	0.76**/0.72**	0.60**/0.56*	0.59**/0.60**
**SAA**	0.42/0.37	0.30/0.30	0.58**/0.37	0.61**/0.51*	0.48*/0.45*
**CRP**	0.33/0.34	0.04/0.11	0.49*/0.35	0.59**/0.43	0.57**/0.55*
**ESR**	0.37/0.48*	0.36/0.35	0.45*/0.42	0.32/0.33	0.35/0.40

*P < 0.05, **P < 0.01

CRP, C-reactive protein; ESR, erythrocyte sedimentation rate; SAA, serum amyloid-A

To assess the validity of the US examination, the 28 joints included in the DAS28 were explored for similarity in detecting arthritis by correlating the number of joints identified by clinical evaluation of swelling (yes vs. no) and BM score (≥ 1 vs. 0). The median (range) correlations were 0.58 (0.43 to 0.85) (*P *= 0.06 to < 0.001). When the sum BM scores of the 28 joints were correlated with the number of joints with clinical swelling, even higher coefficients were found (0.67 (0.48 to 0.87) (*P *= 0.03 to < 0.001).

The median (range) correlation coefficients between the inflammatory biomarkers and assessors global VAS (at the five examinations) were 0.55 (0.37 to 0.74) for calprotectin, 0.57 (0.41 to 0.73) for SAA, 0.59 (0.39 to 0.80) for CRP and 0.45 (0.33 to 0.65) for ESR (correlation coefficients ≥ 0.43 = *P *< 0.05).

Both calprotectin and CRP contributed significantly to explain total sum score BM as the dependent variable in linear regression analyses performed for all examinations during the study (*P *= 0.001 to 0.017 for calprotectin and *P *= 0.012 to 0.038 for CRP), SAA contributed significantly at baseline and six months (*P *= 0.045 and 0.032) and ESR at one and three months (*P *= 0.030 and 0.048). With total sum score PD as the dependent variable, calprotectin contributed significantly at all examinations (*P *= 0.003 to 0.031), CRP contributed significantly at baseline, 6 and 12 months (*P *= 0.001 to 0.026), SAA at 6 months (*P *= 0.002) and ESR at 3 and 6 months (*P *= 0.007 and 0.044).

Calprotectin was found to have higher responsiveness to change than CRP, SAA and ESR (Table 3), with large SRM at 1 month, moderate at 3 and 6 months and small at 12 months examination.

**Table 3:** Standardized response mean (SRM) of the changes from baseline of the four laboratory markers

	SRM 1 month	SRM 3 months	SRM 6 months	SRM 12 months
**Calprotectin**	-0.84	-0.63	-0.63	-0.32
**SAA**	-0.44	-0.55	-0.38	-0.30
**CRP**	-0.10	-0.43	-0.15	-0.12
**ESR**	-0.33	-0.71	-0.03	-0.13

CRP, C-reactive protein; ESR, erythrocyte sedimentation rate; SAA, serum amyloid-A.

## Discussion

To our knowledge, this is the first study to examine the associations between levels of calprotectin and BM/PD scores from a comprehensive US examination. All the inflammatory biomarkers, clinical assessments and US sum scores improved during treatment with adalimumab. Significant correlations were found between the levels of calprotectin and the US scores and the validity of calprotectin as an inflammatory marker was supported by associations with the clinical evaluation of disease activity. In addition, calprotectin was found to have substantial responsiveness to biological treatment.

Calprotectin is released from leukocytes during inflammation. Thus the present objective was to perform an extensive US examination of joints, tendons and bursae to capture the total amount of inflammation. However, the scoring system does not differ between small and large joints. Hence, adjusting for the joint size could have improved the correlations between calprotectin levels and sum US scores. When reduced numbers of joints were analyzed, higher coefficients were found. The strongest correlations were found with the 12-joint score [17], which includes bilateral examination of the elbow, wrist, MCP 2 and 3, knee and ankle in addition to the medial and lateral tendon compartments in the ankle. Thus, in this score most of the joints are relatively large, which may explain the stronger correlations with calprotectin.

The correlations between the laboratory markers and the total sum US scores increased during the study, with highest coefficients between calprotectin and US scores (both comprehensive and reduced number of joints) at the three-month examination (Figure 1). Calprotectin levels have been found to reflect the ongoing inflammation, and at the three-month examination both calprotectin and the US detected synovitis were significantly reduced. Since this is a small study, the different levels of association between calprotectin and US pathology during biologic medication should be explored in larger studies.

Calprotectin is expressed in synovial tissue macrophages localized in the lining layer adjacent to the cartilage-pannus junction [30] and high levels of calprotectin have been found in synovial fluid from RA patients [13]. Calprotectin is suggested to play an active role in the inflammation by amplifying pro-inflammatory cytokine responses via activation of NF-κB and p38 mitogen-activated protein kinase in RA [31]. Calprotectin has been shown to be a reliable marker of inflammation in several rheumatic joint diseases like RA [11,12], juvenile idiopathic arthritis [32,33] and psoriatic arthritis [34]. The present study indicates that calprotectin levels are associated with the overall inflammation in RA patients, including both the amount of synovial hypertrophy explored by BM and the extent of vascularization evaluated by PD.

The regression analyses showed calprotectin to be independently associated with both total sum BM and PD scores, even when DAS28 was included in the equation. Thus, calprotectin was able to give information beyond a clinical composite score. Subclinical inflammation is of major importance in the evaluation of RA patients, and both BM and PD activity have been described in RA patients being in clinical remission [35]. A recent study of patients with juvenile idiopathic arthritis in remission showed that the level of calprotectin was associated with risk of relapse after discontinuing methotrexate [36]. A definition of clinical remission in RA was recently presented by ACR and EULAR [37]. The present finding of associations between calprotectin and the US sum scores suggest that in RA patients a normal calprotectin level could support a clinical evaluation of remission. This important issue should be further explored.

PD activity has been found to be related to the development of joint erosions [38] and we have previously found calprotectin to be associated with radiographic damage [12,14]. Calprotectin plays a major role in the local inflammatory process during RA [9,31], and the possible association between PD activity and calprotectin levels should, therefore, be investigated in a larger study.

The evaluation of US synovitis scores is dependent on the operator. Much work has been done to improve the reliability of US scoring [15-17]. For the joint scores included in the present study there were high intra-reader intra-class correlations for evaluation of stored images (described previously [23]). In addition, the present BM and PD joint scoring was similar to the scoring system used in a US atlas recently applied in a reliability study where we found high intra- and inter- reader reliability [21].

Strengths of the present longitudinal study were that only one experienced sonographer performed the comprehensive US assessments and that all the RA patients used a biologic medication supposed to cause improvement. However, an obvious weakness is the limited number of patients included.

## Conclusions

Calprotectin was found to have high association to US assessments of RA patients, indicating that calprotectin reflects ongoing inflammation. In addition, calprotectin levels decreased during treatment with adalimumab, and had higher sensitivity to change than the traditional inflammatory markers. In contrast to the acute phase proteins being produced in the liver, calprotectin is released from activated leukocytes during inflammation. Our results support that calprotectin may be used as a marker of inflammatory activity in RA patients on biologic treatment.

## Abbreviations

BM: B-mode or grey scale; CRP: C-reactive protein; DAMPs: damage-associated molecular pattern molecules; DAS28: disease activity score (including 28 joints); ESR: erythrocyte sedimentation rate; IL: interleukin; PD: power Doppler activity; RA: rheumatoid arthritis; SAA: serum amyoid A; SRM: standardized response mean; TNF: tumor necrosis factor; US: ultrasonography; VAS: visual analogue scale.

## Competing interests

The authors declare that they have no competing interests.

## Authors' contributions

HBH was the principal investigator for the study, responsible for the study design and performance of all the ultrasonographic examinations as well as writing the manuscript. MKF was responsible for the quantification of calprotectin and giving constructive comments on the manuscript. TNW was responsible for the quantification of serum amyloid A and giving constructive comments on the manuscript. TKK facilitated the performance of the study, giving input to the study design and giving constructive comments on the manuscript. All the authors have read and approved the manuscript for publication.

## References

[B1] van Leeuwen MA Westra J Limburg PC van Riel PL van Rijswijk MH Clinical significance of interleukin-6 measurement in early rheumatoid arthritis: relation with laboratory and clinical variables and radiological progression in a three year prospective study Ann Rheum Dis 1995 54 674 677 10.1136/ard.54.8.674 7677445 PMC1009966

[B2] de Vries MK van Eijk IC van der Horst-Bruinsma IE Peters MJ Nurmohamed MT Dijkmans BA Hazenberg BP Wolbink GJ Erythrocyte sedimentation rate, C-reactive protein level, and serum amyloid a protein for patient selection and monitoring of anti-tumor necrosis factor treatment in ankylosing spondylitis Arthritis Rheum 2009 61 1484 1490 10.1002/art.24838 19877087

[B3] Fagerhol MK Dale I Andersson T A radioimmunoassay for a granulocyte protein as a marker in studies on the turnover of such cells Bull Eur Physiopathol Respir 1980 16 Suppl 273 281 7225633 10.1016/b978-0-08-027379-2.50028-4

[B4] Edgeworth J Gorman M Bennett R Freemont P Hogg N Identification of p8, 14 as a highly abundant heterodimeric calcium binding protein complex of myeloid cells J Biol Chem 1991 266 7706 7713 2019594

[B5] Dale I Brandtzaeg P Fagerhol MK Scott H Distribution of a new myelomonocytic antigen (L1) in human peripheral blood leukocytes Am J Clin Pathol 1985 84 24 34 2409791 10.1093/ajcp/84.1.24

[B6] Andersson KB Sletten K Berntzen HB Dale I Brandtzaeg P Jellum E Fagerhol MK The leucocyte L1 protein: Identity with the cystic fibrosis antigen and the calcium-binding MRP-8 and MRP-14 macrophage components Scand J Immunol 1988 28 241 245 10.1111/j.1365-3083.1988.tb02437.x 3413449

[B7] Odink K Cerletti N Brüggen J Clerc RG Tarcsay L Zwadlo G Gerhards G Schlegel R Sorg C Two calcium-binding proteins in infiltrate macrophages of rheumatoid arthritis Nature 1987 330 80 82 10.1038/330080a0 3313057

[B8] Foell D Roth J Proinflammatory S100 proteins in arthritis and autoimmune disease Arthritis Rheum 2004 50 3762 3771 10.1002/art.20631 15593206

[B9] Loser K Vogl T Voskort M Lueken A Kupas V Nacken W Klenner L Kuhn A Foell D Sorokin L Luger TA Roth J Beissert S The Toll-like receptor 4 ligands Mrp8 and Mrp14 are crucial in the development of autoreactive CD8+ T cells Nat Med 2010 16 713 717 10.1038/nm.2150 20473308

[B10] Johne B Fagerhol MK Lyberg T Prydz H Brandtzaeg P Naess-Andresen CF Dale I Functional and clinical aspects of the myelomonocyte protein calprotectin J Clin Pathol: Mol Pathol 1997 50 113 123 10.1136/mp.50.3.113 PMC379605 9292145

[B11] Berntzen HB Munthe E Fagerhol MK A longitudinal study of the leukocyte protein L1 as an indicator of disease activity in patients with rheumatoid arthritis J Rheumatol 1989 16 1416 1420 2600939

[B12] Hammer HB Odegard S Fagerhol MK Landewé R van der Heijde D Uhlig T Mowinckel P Kvien TK Calprotectin (a major leucocyte protein) is strongly and independently correlated with joint inflammation and damage in rheumatoid arthritis Ann Rheum Dis 2007 66 1093 1097 10.1136/ard.2006.064741 17234650 PMC1954700

[B13] Berntzen HB Ölmez Ü Fagerhol MK Munthe E The leukocyte protein L1 in plasma and synovial fluid from patients with rheumatoid arthritis and osteoarthritis Scand J Rheumatol 1991 20 74 82 10.3109/03009749109165280 1709519

[B14] Hammer HB Ødegård S Syversen SW Landewé R van der Heijde D Uhlig T Mowinckel P Kvien TK Calprotectin (a major S100 leukocyte protein) predicts 10-year radiographic progression in patients with rheumatoid arthritis Ann Rheum Dis 2010 69 150 154 10.1136/ard.2008.103739 19095696

[B15] Naredo E Bonilla G Gamero F Uson J Carmona L Laffon A A Assessment of inflammatory activity in rheumatoid arthritis: a comparative study of clinical evaluation with grey scale and power Doppler ultrasonography Ann Rheum Dis 2005 64 375 381 15708891 10.1136/ard.2004.023929 PMC1755396

[B16] Szkudlarek M Klarlund M Narvestad E Court-Payen M Strandberg C Jensen KE Thomsen HS Østergaard M Ultrasonography of the metacarpophalangeal and proximal interphalangeal joints in rheumatoid arthritis: a comparison with magnetic resonance imaging, conventional radiography and clinical examination Arthritis Res Ther 2006 8 R52 10.1186/ar1904 16519793 PMC1526591

[B17] Naredo E Rodríguez M Campos C Rodríguez-Heredia JM Medina JA Giner E Martínez O Toyos FJ Ruíz T Ros I Pujol M Miquel X García L Aznar JJ Chamizo E Páez M Morales P Rueda A Tuneu R Corominas H de Agustín JJ Moragues C Mínguez D Willisch A González-Cruz I Aragón A Iglesias G Armas C Pablo Valdazo J Vargas C Validity, reproducibility, and responsiveness of a twelve-joint simplified power doppler ultrasonographic assessment of joint inflammation in rheumatoid arthritis Arthritis Rheum 2008 59 515 522 10.1002/art.23529 18383408

[B18] Terslev L Torp-Pedersen S Qvistgaard E Kristoffersen H Røgind H Danneskiold-Samsøe B Bliddal H Effects of treatment with etanercept (Enbrel, TNRF:Fc) on rheumatoid arthritis evaluated by Doppler ultrasonography Ann Rheum Dis 2003 62 178 181 10.1136/ard.62.2.178 12525391 PMC1754421

[B19] Filippucci E Iagnocco A Salaffi F Cerioni A Valesini G Grassi W Power Doppler sonography monitoring of synovial perfusion at the wrist joints in patients with rheumatoid arthritis treated with adalimumab Ann Rheum Dis 2006 65 1433 1437 10.1136/ard.2005.044628 16504996 PMC1798349

[B20] Iagnocco A Filippucci E Perella C Ceccarelli F Cassarà E Alessandri C Sabatini E Grassi W Valesini G Clinical and ultrasonographic monitoring of response to adalimumab treatment in rheumatoid arthritis J Rheumatol 2008 35 35 40 18050384

[B21] Hammer HB Bolton-King P Bakkeheim V Berg TH Sundt E Kongtorp AK Haavardsholm EA Examination of intra-and inter-rater reliability with a new ultrasonographic reference atlas for scoring of synovitis in patients with rheumatoid arthritis Ann Rheum Dis 2011 70 1995 1998 10.1136/ard.2011.152926 21784724

[B22] Hammer HB Kvien TK Comparisons of 7- to 78-joint ultrasonography scores: all different joint combinations show equal response to adalimumab treatment in patients with rheumatoid arthritis Arthritis Res Ther 2011 13 R78 10.1186/ar3341 21619619 PMC3218888

[B23] Hammer HB Sveinsson M Kongtorp AK Kvien TK A 78-joints ultrasonographic assessment is associated to clinical assessments and is highly responsive to improvement in a longitudinal study of patients with rheumatoid arthritis starting adalimumab treatment Ann Rheum Dis 2010 69 1349 1351 10.1136/ard.2009.126995 20472599

[B24] Arnett FC Edworthy SM Bloch DA McShane DJ Fries JF Cooper NS Healey LA Kaplan SR Liang MH Luthra HS The American Rheumatism Association 1987 revised criteria for the classification of rheumatoid arthritis Arthritis Rheum 1988 31 315 324 10.1002/art.1780310302 3358796

[B25] Ledue TB Weiner DL Sipe JD Poulin SE Collins MF Rifai N Analytical evaluation of particle-enhanced immunonephelometric assays for C-reactive protein, serum amyloid A and mannose-binding protein in human serum Ann Clin Biochem 1998 35 745 753 9838988 10.1177/000456329803500607

[B26] Røseth AG Fagerhol MK Aadland E Schjønsby H Assessment of the neutrophil dominating protein calprotectin in feces. A methodologic study Scand J Gastroenterol 1992 27 793 798 10.3109/00365529209011186 1411288

[B27] Kristinsson J Røseth A Fagerhol MK Aadland E Schjønsby H Børmer OP Raknerud N Nygaard K Fecal calprotectin concentration in patients with colorectal carcinoma Dis Colon Rectum 1998 41 316 321 10.1007/BF02237485 9514426

[B28] Prevoo ML van't Hof MA Kuper HH van Leeuwen MA van de Putte LB van Riel PL Modified disease activity scores that include twenty-eight-joint counts. Development and validation in a prospective longitudinal study of patients with rheumatoid arthritis Arthritis Rheum 1995 38 44 48 10.1002/art.1780380107 7818570

[B29] Backhaus M Burmester GR Gerber T Grassi W Machold KP Swen WA Wakefield RJ Manger B Guidelines for musculoskeletal ultrasound in rheumatology. Working Group for Musculoskeletal Ultrasound in the EULAR Standing Committee on International Clinical Studies including Therapeutic Trials Ann Rheum Dis 2001 60 641 649 10.1136/ard.60.7.641 11406516 PMC1753749

[B30] Youssef P Roth J Frosch M Costello P Fitzgerald O Sorg C Bresnihan B Expression of myeloid related proteins (MRP) 8 and 14 and the MRP8/14 heterodimer in rheumatoid arthritis synovial membrane J Rheumatol 1999 26 2523 2528 10606357

[B31] Sunahori K Yamamura M Yamana J Takasugi K Kawashima M Yamamoto H Chazin WJ Nakatani Y Yui S Makino H The S100A8/A9 heterodimer amplifies proinflammatory cytokine production by macrophages via activation of nuclear factor kappa B and p38 mitogen-activated protein kinase in rheumatoid arthritis Arthritis Res Ther 2006 8 R69 10.1186/ar1939 16613612 PMC1526633

[B32] Berntzen HB Fagerhol MK Østensen M Mowinckel P Høyeraal HM The L1 protein as a new indicator of inflammatory activity in patients with juvenile rheumatoid arthritis J Rheumatol 1991 18 133 138 2023183

[B33] Frosch M Strey A Vogl T Wulffraat NM Kuis W Sunderkötter C Harms E Sorg C Roth J Myeloid-related proteins 8 and 14 are specifically secreted during interaction of phagocytes and activated endothelium and are useful markers for monitoring disease activity in pauciarticular-onset juvenile rheumatoid arthritis Arthritis Rheum 2000 43 628 637 10.1002/1529-0131(200003)43:3<628::AID-ANR20>3.0.CO;2-X 10728757

[B34] Kane D Roth J Frosch M Vogl T Bresnihan B FitzGerald O Increased perivascular synovial membrane expression of myeloid-related proteins in psoriatic arthritis Arthritis Rheum 2003 48 1676 1685 10.1002/art.10988 12794836

[B35] Brown AK Quinn MA Karim Z Conaghan PG Peterfy CG Hensor E Wakefield RJ O'Connor PJ Emery P Presence of significant synovitis in rheumatoid arthritis patients with disease-modifying antirheumatic drug-induced clinical remission: evidence from an imaging study may explain structural progression Arthritis Rheum 2006 54 3761 3773 10.1002/art.22190 17133543

[B36] Foell D Wulffraat N Wedderburn LR Wittkowski H Frosch M Gerss J Stanevicha V Mihaylova D Ferriani V Tsakalidou FK Foeldvari I Cuttica R Gonzalez B Ravelli A Khubchandani R Oliveira S Armbrust W Garay S Vojinovic J Norambuena X Gamir ML García-Consuegra J Lepore L Susic G Corona F Dolezalova P Pistorio A Martini A Ruperto N Roth J Methotrexate withdrawal at 6 vs 12 months in juvenile idiopathic arthritis in remission: a randomized clinical trial JAMA 2010 303 1266 1273 10.1001/jama.2010.375 20371785

[B37] Felson DT Smolen JS Wells G Zhang B van Tuyl LH Funovits J Aletaha D Allaart CF Bathon J Bombardieri S Brooks P Brown A Matucci-Cerinic M Choi H Combe B de Wit M Dougados M Emery P Furst D Gomez-Reino J Hawker G Keystone E Khanna D Kirwan J Kvien TK Landewé R Listing J Michaud K Martin-Mola E Montie P American College of Rheumatology/European League Against Rheumatism provisional definition of remission in rheumatoid arthritis for clinical trials Ann Rheum Dis 2011 70 404 413 10.1136/ard.2011.149765 21292833

[B38] Naredo E Möller I Cruz A Carmona L Carrido J Power Doppler ultrasonographic monitoring of response to anti-tumor necrosis factor therapy in patients with rheumatoid arthritis Arthritis Rheum 2008 58 2248 2256 10.1002/art.23682 18668537

